# Detecting and Locating the Site of Local Relapse Using ^18^F-PSMA-1007 Imaging After Primary Treatment of 135 Prostate Cancer Patients—Potential Impact on PSMA-Guided Radiation Therapy

**DOI:** 10.1007/s11307-022-01766-6

**Published:** 2022-08-23

**Authors:** S. A. Koerber, R. C. Kroener, K. Dendl, C. Kratochwil, C. A. Fink, J. Ristau, E. Winter, K. Herfarth, G. Hatiboglu, M. Hohenfellner, U. Haberkorn, J. Debus, F. L. Giesel

**Affiliations:** 1 Department of Radiation Oncology, Heidelberg University Hospital, Im Neuenheimer Feld 400, 69120 Heidelberg, Germany; 2 National Center for Tumor Diseases (NCT), Im Neuenheimer Feld 460, 69120 Heidelberg, Germany; 3 Heidelberg Institute of Radiation Oncology (HIRO), Im Neuenheimer Feld 400, 69120 Heidelberg, Germany; 4 Clinical Cooperation Unit Radiation Oncology, German Cancer Research Center (DKFZ), Im Neuenheimer Feld 280, 69120 Heidelberg, Germany; 5 Department of Nuclear Medicine, Heidelberg University Hospital, Im Neuenheimer Feld 400, 69120 Heidelberg, Germany; 6 Clinical Cooperation Unit Nuclear Medicine, German Cancer Research Center (DKFZ), Im Neuenheimer Feld 280, 69120 Heidelberg, Germany; 7 Heidelberg Ion-Beam Therapy Center (HIT), Department of Radiation Oncology, Heidelberg University Hospital, Im Neuenheimer Feld 450, 69120 Heidelberg, Germany; 8 Department of Urology, Heidelberg University Hospital, Im Neuenheimer Feld 110, 69120 Heidelberg, Germany; 9 German Cancer Consortium (DKTK), partner site Heidelberg, Im Neuenheimer Feld 280, 69120 Heidelberg, Germany; 10 Department of Nuclear Medicine, Medical Faculty, Heinrich-Heine-University, University Hospital Duesseldorf, Moorenstr. 5, Duesseldorf, Germany

**Keywords:** Prostate cancer, PSMA, PET/CT, 18F-PSMA-1007, Local relapse

## Abstract

**Purpose:**

Due to limited imaging options, the visualization of a local relapse of prostate cancer used to pose a considerable challenge. However, since the integration of ^18^F-PSMA-1007-PET/CT into the clinic, a relapsed tumor can now easily be detected by hybrid imaging. The present study aimed to evaluate and map the allocate relapse in a large cohort of prostate cancer patients focusing on individual patient management conclusions for radiation therapy.

**Procedures:**

The current study included 135 men with prostate cancer after primary treatment who underwent ^18^F-PSMA-1007-PET/CT due to biochemical relapse detecting a local relapse. Imaging data were reassessed and analyzed with regard to relapse locations. For the correlation of tumor foci with clinical data, we used binary logistic regression models as well as the Kruskal–Wallis test and Mann–Whitney test.

**Results:**

In total, 69.6% of all patients (mean age: 65 years) underwent prostatectomy while 30.4% underwent radiation therapy. PET imaging detected most frequently a unifocal relapse (72.6%). There was a statistically significantly higher rate of ipsilateral cases among the relapsed tumors. Comparing both treatment approaches, tumors relapsed most commonly within the posterior region after surgery and transition/peripheral zone after radiation therapy, respectively.

**Conclusions:**

The present study confirms that ^18^F-PSMA-1007-PET/CT is highly suitable for the localization and allocation of a local relapse in patients with prostate cancer. The data enable further optimizing dose prescriptions and target volume delineations of radiation therapy in the future.

**Supplementary Information:**

The online version contains supplementary material available at 10.1007/s11307-022-01766-6.

## Background

Due to its limited expression in extraprostatic tissue and the upregulated expression in many malignant prostate lesions, prostate-specific membrane antigen (PSMA) as a target is highly suitable for theranostics in the era of modern personalized oncology [1–3]. To date, several ligands targeting PSMA—also known as glutamate carboxypeptidase II—have been developed and are used for imaging and therapy of patients with prostate cancer. Conjugated with galium-68 and flourine-18, which are the most commonly used tracers, PSMA expression can be imaged with a high sensitivity and specificity [4, 5]. Since 2011, numerous clinical trials have evaluated the role of PSMA-PET/CT as an imaging tool focusing on the role as a restaging probe. In one of the largest evaluations of a retrospective study including 2533 patients with recurrent prostate cancer after prostatectomy, Afshar-Oromieh reported on a very promising performance of ^68^ Ga-PSMA-11 PET/CT. Pathologic PET/CT scans were observed in 43%, 58%, and 72% of men with PSA ≤ 0.2, > 0.2 to ≤ 0.5, and > 0.5 to ≤ 1.0 ng/ml, respectively [6]. Similar results were obtained by a prospective multi-center study in 2005 patients: The use of ^68^ Ga-PSMA-11 as a radiotracer leads to a high detection rate in this large cohort of men with biochemical relapse after surgery. The authors observed positive findings in 44.8% (PSA < 0.25 ng/ml) to 96.2% (PSA > 10 ng/ml), significantly increasing with rising PSA levels [7]. Although ^68^ Ga-PSMA-11 has a favorable tumor-to-background ratio and a high accuracy, the tracer is excreted by the urinary tract [8, 9]. Thus, the detection of a local relapse after primary treatment is challenging.

Since the introduction of a novel tracer, ^18^F-PSMA-1007, restaging after biochemical relapse is highly efficient due to a very low renal excretion tract minimizing the risk of inconclusive results within the prostate bed [10]. In a cohort of 251 patients from three academic centers, ^18^F-PSMA-1007-PET/CT demonstrated a high detection rate for men with biochemical relapse after surgery which might improve patient management by correctly identifying sites of relapse at an early stage [11]. Therefore, the purpose of this study was to evaluate the role of PSMA imaging, using ^18^F-PSMA-1007 as a tracer, in characterizing and mapping the local relapse in a large cohort of prostate cancer patients after prostatectomy or definitive radiation therapy. Our results may contribute to more individualized salvage strategies or improvements with regard to primary treatment.

## Materials and Methods

### Study Design

For this mono-centric study, we initially identified more than 2000 patients from our database who underwent PSMA-PET/CT for restaging due to a biochemical relapse after primary treatment between June 2011 and April 2019. All patients gave written consent to undergo the imaging procedures. From these patients, 519 men with local relapse in hybrid imaging were identified. Finally, 135 men met the inclusion criteria (age of 18 or older, sufficient clinical data, PET imaging with ^18^F-PSMA-1007) and were included in the present analysis. Clinical parameters such as initial PSA, Gleason score, and initial tumor location were anonymously collected. The tumor relapse location for each patient was identified by reassessment of ^18^F-PSMA-1007-PET/CT and transferred to a template, thus allowing further analysis of relapse locations with regard to clinical subgroups. A map was created showing infiltrated areas and predominant relapse locations in the prostatic fossa.

This present retrospective study was approved by the local Institutional Review Board (“S-433/2019”) and conducted in accordance with the Helsinki Declaration and its amendments.

### Imaging Protocol and Image Analysis

Imaging was performed according to local standard protocols using Siemens mCT flow, Siemens Biograph 6, and Siemens Biograph 20 mCT scanners 90–120 min after application of ^18^F-PSMA-1007. An effective dose of approximately 4.4–5.5 mSv per 200–250 MBq examination was applied [10, 12]. For image evaluation, we used the “syngo TrueD” software (Siemens Healthineers, Erlangen/Germany). Maximum standardized uptake values (SUV_max_) were quantified via semi-automatic regions of interest (ROI). The images were evaluated by two certified nuclear physicians as well as one certified radiation oncologist. We classified any tracer accumulation that did not correspond to a physiological uptake as malignant. All these findings were collected in a common consensus.

The location of tumor relapse after radical prostatectomy was evaluated by allocating each tumor to a template of the prostatic fossa consisting of 27 regions (SUPPLEMENT 1). Tumor locations in patients who received definitive radiation therapy were allocated using the PIRADS 2.1 template [13]. A map was created showing the region-specific incidence within the prostatic fossa which was infiltrated. Summation images indicate the predominant locations of tumor relapse. The number of local tumor foci was evaluated in each patient. For subgroup analysis, the type of primary definitive treatment (radiation therapy, surgery), surgical technique (non-robotic-assisted, robotic-assisted), and location of the primary tumor prior to definitive treatment were identified in the database. The primary tumor location was categorized into “left side,” “right side,” and “both sides”.

### Statistical Analysis

We used Microsoft Excel and SPSS Statistics version 27 (IBM, Armonk/NY, USA) for statistical analysis. After descriptive evaluation, the initial 27 regions were aggregated into a total of 7 regions to make them feasible for further statistical analysis using binary logistic regression models in order to investigate correlations between tumor location and clinical parameters. Tumor infiltration in either the right or the left prostate lobe, in the anterior or posterior region, and in close proximity to the bladder, bladder neck, or anastomotic site as well as mono-focal or bi-/multifocal tumor relapse served as dependent variables. Independent variables included “type of definitive treatment” (surgery or radiation therapy), “primary tumor location” (left or right prostate lobe—including seminal vesicles—or both sides), and “surgical technique” (non-robotic-assisted or robotic-assisted). Further analysis on the amount of tumor foci in relation to the type of primary definitive treatment was performed using non-parametric testing (Kruskal–Wallis test and Mann–Whitney test). A *p*-value below 0.05 was considered statistically significant.

## Results

### Cohort Characteristics

In total, 135 men with prostate cancer and a first biochemical relapse were included in the current study. All patients had a histologically confirmed carcinoma after biopsy and underwent prostatectomy (*n* = 94; 69.6%) or radiation therapy (*n* = 41; 30.4%) as primary treatment. Most patients received robotic surgery (48.1%), and 22 patients underwent retropubic prostatectomy. Intensity-modulated radiation therapy (IMRT) with photons was performed for almost 52% of all irradiated patients, and other techniques included a combination of photon and proton irradiation (13.8%), brachytherapy (24.1%), carbon ions (6.9%), and protons (3.4%).

Mean age of the cohort was 65 years (range: 47–82 years; standard deviation [SD]: 7) and most tumors (46.3%) were classified as high risk according to d’Amico risk classification [14]. All patients received ^18^F-PSMA-1007 PET/CT imaging due to biochemical relapse at a mean PSA level of 10.66 ng/ml (range: 0.08–720; SD: 65.05). Table 1 summarizes the patient characteristics of the entire cohort.

**Table 1:** Patient characteristics

Total number of patients	*n* = *135*
Age at PSMA-PET/CT [years], mean (range; standard deviation)	65 (47–82; 7)
Gleason score, *n* = *106*	
5	2 (1.9%)
6	10 (9.4%)
7	61 (57.5%)
8	13 (12.3%)
9	19 (17.9%)
10	1 (0.9%)
PSA at initial diagnosis [ng/ml], mean (range; standard deviation), *n* = *97*	27 (0.2–444; 57.99)
< 10	51 (52.6%)
10–20	21 (21.6%)
> 20	25 (25.8%)
Risk classification according d’Amico, *n* = *108*	
Low risk	5 (4.6%)
Intermediate risk	17 (15.7%)
High risk	50 (46.3%)
Unknown (intermediate or high risk)	36 (33.3%)
PSA at [18]F-PSMA-PET-CT [ng/ml], mean (range; standard deviation), *n* = *125*	2.15 (0.08–720; 65.05)

### Imaging Results

**Fig. 1:**
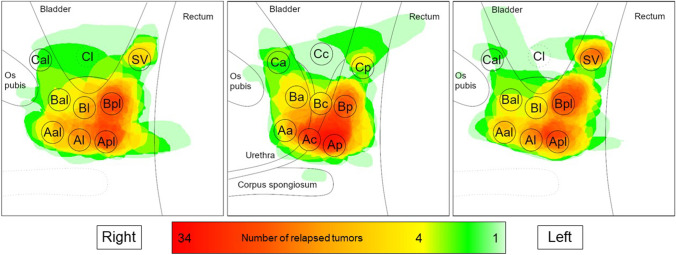
Map of local relapses after surgery according to frequency (*n* = 94)

**Fig. 2:**
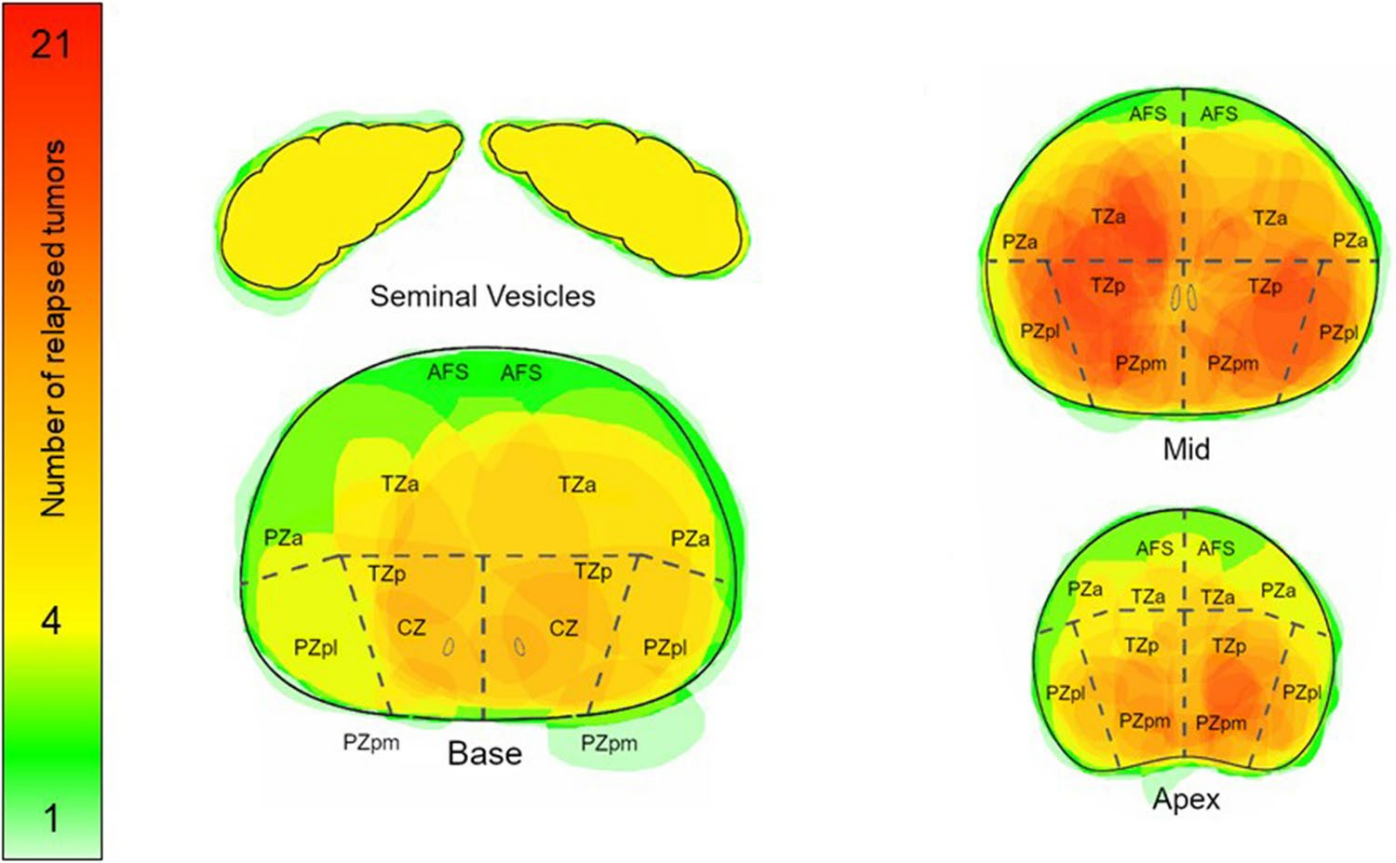
Map of local relapses after radiation therapy according to frequency (*n* = 41)

Most local relapses detected by PSMA-PET/CT imaging were unifocal (72.6%). Hybrid imaging resulted in 23.0% bifocal relapsed tumors, while more than two foci could be identified in 4.5% of the cohort. Bifocal or multifocal tumor infiltration was particularly frequent among patients who received primary radiation therapy (43.9%) compared to 20.2% in patients who underwent surgery (*p* = 0.005). For all patients included in the trial, the aspect ratio of all local relapses was well balanced with an infiltration rate of 62.2% (right) compared to 57.8% (left). In general, there was a higher rate of relapses in the posterior region (94.8%). Detailed region-specific incidence of the prostate and the prostatic fossa as well as standardized uptake values are displayed in Figs. 1 and 2 and Table 2.

**Table 2:** Standardized uptake values of all local relapses with regard to tumor location

Relapse after surgery, *n* = *117*	SUV_max_: mean (standard deviation)	SUV_max_: range
Anastomotic site	13.04 (12.04)	2.46–56.18
Bladder neck	16.76 (13.46)	3.17–73.84
Bladder	16.76 (15.38)	2.47–46.00
Seminal vesical	12.59 (12.00)	4.34–45.00
relapse after radiation therapy, *n* = *65*	SUV_max_: mean (standard deviation)	SUV_max_ mean (range)
Transitional zone	11.44 (10.44)	3.79–50.12
Peripheral zone	12.78 (11.44)	3.79–50.12
Central zone	10.31 (17.30)	4.18–50.12
Anterior fibromuscular stroma (AFS)	21.67 (16.46)	5.66–50.12
Seminal vesicles	22.63 (15.26)	6.11–40.90

A comparison of PSMA-PET/CT with imaging data obtained before primary treatment demonstrated that a local relapse has a similar aspect ratio like the primary tumor site. Prostate carcinomas on the right led to a significantly more frequent relapse on the right (*p* = 0.025). Although the left side did not reach statistical significance, there was also a trend towards an ipsilateral occurrence (*p* = 0.167).

### Subgroup Analyses

The common location of relapse for patients who underwent prostatectomy was an infiltration of the posterior region (89.4%). An infiltration rate of 25.5%, 47.9%, and 66.0% was observed for the bladder region, bladder neck, and anastomotic site, respectively (Fig. 1). The binary logistic regression model confirmed the increased risk for an ipsilateral relapse in the prostate bed (*p* = 0.040/0.032). Testing different hypotheses for subgroup analyses, we only noted a trend towards a higher rate of relapse within the site of anastomosis after Da Vinci surgery compared to other techniques (*p* = 0.068) (Table 3).

**Table 3:** Subgroup Characteristics

Total number of patients who underwent surgery	*n* = *94*
Tumor infiltration in selected regions (non-exclusive), *n* = *94*	
Anterior	27 (28.4%)
Urethral/central bladder	39 (41.1%)
Posterior	85 (89.5%)
Bladder region	23 (24.2%)
Bladder neck	45 (47.4%)
Anastomotic site	62 (65.3%)
Infiltrated side for tumors who were initially located in the right side, *n* = *8*	
Right side	7 (87.5%)
Left side	1 (12.5%)
Exclusively right side	6 (75.0%)
Exclusively left side	0 (0.0%)
Infiltrated side for tumors who were initially located in the left side, *n* = *9*	
Right side	2 (22.2%)
Left side	7 (77.8%)
Exclusively right side	0 (0.0%)
Exclusively left side	4 (44.4%)
Infiltrated side for tumors who were initially located both sides, *n* = *19*	
Right side	6 (31.6%)
Left side	7 (36.8%)
Exclusively right side	4(21.1%)
Exclusively left side	2 (10.5%)
Tumor infiltration in selected regions after primary retropubic non-robotic-assisted surgery, *n* = *22*	
Bladder and seminal vesicles	6 (27.3%)
Bladder neck	14 (63.6%)
Anastomotic site	9 (40.9%)
Exclusively anastomotic site	4 (18.2%)
Tumor infiltration in selected regions after primary robotic-assisted surgery, *n* = *25*	
Bladder and seminal vesicles	5 (20.0%)
Bladder neck	9 (36.0%)
Anastomotic site	18 (72.0%)
Exclusively anastomotic site	12 (48.0%)
Total number of patients who underwent primary definitive radiation therapy	*n* = *41*
Tumor infiltration in selected regions (non-exclusive), *n* = *41*	
Transitional zone	34 (82.9%)
Peripheral zone	34 (82.9%)
Anterior fibromuscular stroma	11 (26.8%)
Central zone	12 (29.3%)
Seminal vesicles	7 (17.1%)
Anterior zones	29 (70.7%)
Posterior zones	39 (95.1%)
Exclusively anterior zones	2 (4.9%)
Exclusively posterior zones	12 (29.3%)
Right side	40 (97.6%)
Left side	32 (78.0%)
Exclusively right side	9 (22.0%)
Exclusively left side	1 (2.4%)

Within the cohort who underwent definitive radiation therapy, a local relapse infiltrated most frequently the transition zone (82.9%) and peripheral zone (82.9%). An infiltration of the seminal vesicle region was quite rare and was only observed in 7 patients. A total of 70.7% and 95.1% of all relapses infiltrated the anterior region or the posterior region, respectively (Fig. 2). No statistically significant difference with regard to the site of recurrence was observed concerning irradiation technique (Table 3).

## Discussion

Our data demonstrated that ^18^F-PSMA-1007 PET/CT is able to reliably detect a local relapse of prostate cancer after primary treatment. Due to its biodistribution, the radioligand is well suited for identifying and characterizing tumor tissue within the prostatic fossa. Although minor unspecific uptake can be observed (e.g., within the skeleton), ^18^F-PSMA-1007 PET/CT may hereby confirm its major role as an imaging probe for restaging [15–17]. Considering the superiority of PSMA imaging when compared to conventional morphological staging such as CT or MRI [18–20], the present analysis provides fundamental data with regard to local recurrence using molecular imaging probes.

**Fig. 3:**
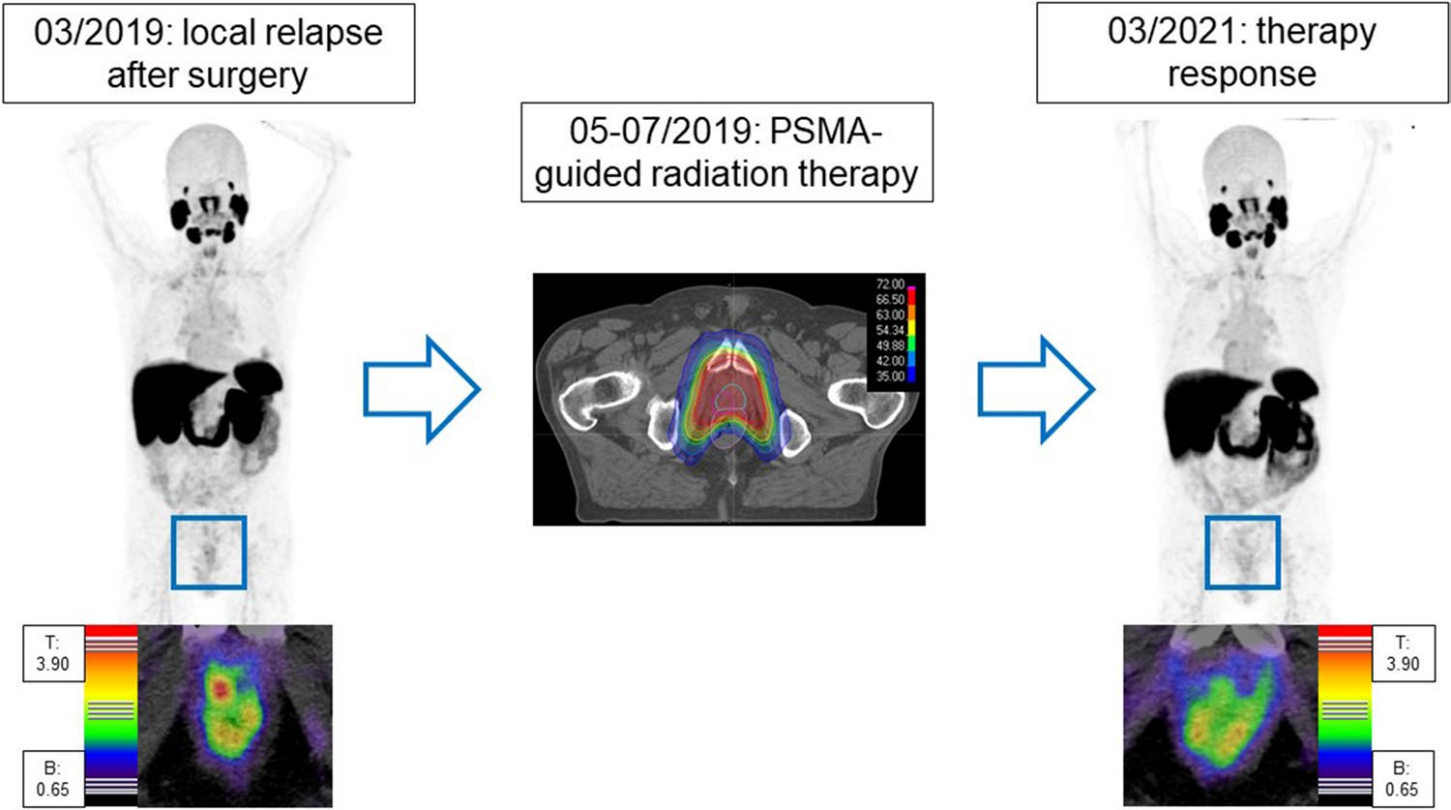
An 80-year-old patient with PSMA-positive local relapse of a high-risk prostate cancer (**a**, **d**) undergoing salvage radiation therapy with simultaneous integrated boost (**b**, **e**). Imaging almost 3 years later showed excellent local control (**c**, **f**)

After surgery, the most frequent site of recurrence for our cohort was the perianastomotic and posterior region. This is in accordance with data obtained from multiparametric MRI (mpMRI), where a relapse was found predominantly at the perianastomotic site or retrovesical: From 70 patients with prostate cancer undergoing mpMRI due to biochemical recurrence, 20 carcinomas relapsed within these areas [21]. Moreover, Liauw et al. observed in a similar cohort that the most frequent locations of recurrence were perianastomotic (67%) or retrovesical (33%) [22]. For adjuvant or salvage radiation therapy, these results are of great interest with regard to target volume delineation. To date, there is an increasing trend towards minimizing safety margins leading to an optimized organ at risk (OAR) sparing [23, 24]. Especially when using modern radiotherapy techniques such as protons or carbon ions with a characteristically sharp dose application while sparing the surrounding healthy tissue [25], the reduction of safety margins should be exercised with a call for caution considering the close proximity of the detected relapses to neighboring organs such as the bladder or rectum. Image-guided radiation therapy (IGRT) with the routine use of CT or MRI scans may avoid an overzealous OAR sparing leading to a relevant loss of dose within the relapsed tumor region. Moreover, future trials should address a local dose escalation of postoperative radiotherapy due to the high rate of ipsilateral relapses. As previously demonstrated in the FLAME trial for the definitive concept, the addition of a focal boost results in an improved biochemical disease-free survival (bDFS) for patients with intermediate and high-risk prostate cancer [26]. Thus, patients who already underwent surgery may benefit from a focal dose escalation within the visible tumor as well (Fig. 3).

Similar conclusions can be postulated for radiation therapy as a primary treatment approach. The FLAME trial also observed a decreasing risk of biochemical failure by increasing the dose of the gross tumor volume (GTV) [26]. Therefore, focal dose escalation strategies may be helpful in improving local control for patients undergoing primary radiation therapy without additional toxicity. Due to a relatively low rate of relapses within the seminal vesicle region for our cohort, international recommendations for the delineation of clinical target volume for prostate cancer stratifying the inclusion of the seminal vesicles according to risk classification seem to be efficient [27]. The adherence to established contouring guidelines may also offer an explanation for the lack of side-specific recurrence with regard to the applied irradiation technique—in contrast to patients who underwent radical prostatectomy. Interestingly, there was a trend towards a higher rate of perianastomotic relapse after robotic-assisted prostatectomy compared to other surgical techniques. Higher rates of nerve-sparing-surgery or preservation of the bladder neck might explain that trend and will be evaluated within future analyses.

Although the present study is—to the best of our knowledge—the largest evaluating and mapping a local relapse of prostate cancer after primary treatment using ^18^F-PSMA-1007-PET/CT, it has several limitations. First, our investigation obtained data from only one institution and had a retrospective design, which can result in patient selection bias. Due to different treatment approaches, we used a heterogenous classification of relapse complicating a detailed evaluation of the entire cohort. Moreover, subgroup analyses of irradiated patients should be interpreted with caution due to the relatively low number of patients who underwent primary radiotherapy. Nevertheless, the present study was able to confirm the important role of ^18^F-PSMA-1007-PET/CT as a precise molecular restaging imaging probe to allocate prostate cancer and local relapse. Prospective trials are needed to evaluate the role of local dose escalation strategies or individual adapted target volume delineation for radiation therapy.

## Conclusion

PET/CT using ^18^F-PSMA-1007 as a tracer is highly efficient in detecting a local relapse of prostate cancer after prostatectomy or radiation therapy. The current data may be helpful in optimizing future irradiation strategies for both primary and adjuvant/salvage radiation therapy.

## Supplementary Information

Below is the link to the electronic supplementary material.

Supplementary file1 (DOCX 99 KB)

## References

[1] Sokoloff RL, Norton KC, Gasior CL (2000). A dual-monoclonal sandwich assay for prostate-specific membrane antigen: levels in tissues, seminal fluid and urine. Prostate.

[2] Ross JS, Sheehan CE, Fisher HA (2003). Correlation of primary tumor prostate-specific membrane antigen expression with disease recurrence in prostate cancer. Clin Cancer Res.

[3] Perner S, Hofer MD, Kim R (2007). Prostate-specific membrane antigen expression as a predictor of prostate cancer progression. Hum Pathol.

[4] Bednarova S, Lindenberg ML, Vinsensia M (2017). Positron emission tomography (PET) in primary prostate cancer staging and risk assessment. Transl Androl Urol.

[5] Eiber M, Fendler WP, Rowe SP (2017). Prostate-specific membrane antigen ligands for imaging and therapy. J Nucl Med.

[6] Afshar-Oromieh A, da Cunha ML, Wagner J (2021). Performance of ^68^Ga-PSMA-11 PET/CT in patients with recurrent prostate cancer after prostatectomy-a multi-centre evaluation of 2533 patients. Eur J Nucl Med Mol Imaging.

[7] Abghari-Gerst M, Armstrong WR, Nguyen K (2022). A comprehensive assessment of ^68^Ga-PSMA-11 PET in biochemically recurrent prostate cancer: results from a prospective multicenter study on 2,005 patients. J Nucl Med.

[8] Afshar-Oromieh A, Malcher A, Eder M (2013). PET imaging with a ^68^Gallium-labelled PSMA ligand for the diagnosis of prostate cancer: biodistribution in humans and first evaluation of tumour lesions. Eur J Nucl Med Mol Imaging.

[9] Eder M, Neels O, Muller M (2014). Novel preclinical and radiopharmaceutical aspects of ^68^Ga-PSMA-HBED-CC: a new PET tracer for imaging of prostate cancer. Pharmaceuticals (Basel).

[10] Giesel FL, Hadaschik B, Cardinale J (2017). F-18 labelled PSMA-1007: biodistribution, radiation dosimetry and histopathological validation of tumor lesions in prostate cancer patients. Eur J Nucl Med Mol Imaging.

[11] Giesel FL, Knorr K, Spohn F (2019). Detection efficacy of ^18^F-PSMA-1007 PET/CT in 251 patients with biochemical recurrence of prostate cancer after radical prostatectomy. J Nucl Med.

[12] Wondergem M, van der Zant FM, Knol RJJ (2017). ^18^F-DCFPyL PET/CT in the detection of prostate cancer at 60 and 120 minutes: detection rate, image quality, activity kinetics, and biodistribution. J Nucl Med.

[13] Turkbey B, Rosenkrantz AB, Haider MA (2019). Prostate imaging reporting and data system version 2.1: 2019 update of prostate imaging reporting and data system version 2. Eur Urol.

[14] D’Amico AV, Whittington R, Malkowicz SB (1998). Biochemical outcome after radical prostatectomy, external beam radiation therapy, or interstitial radiation therapy for clinically localized prostate cancer. JAMA.

[15] Arnfield EG, Thomas PA, Roberts MJ (2021). Clinical insignificance of [18F]PSA-1007 avid non-specific bone lesions: a retrospective evaluation. Eur J Nucl Med Mol Imaging.

[16] Rahbar K, Afshar-Oromieh A, Seifert R (2018). Diagnostic performance of ^18^F-PSMA-1007 PET/CT in patients with biochemical recurrent prostate cancer. Eur J Nucl Med Mol Imaging.

[17] AhmadiBidakhvidi N, Laenen A, Jentjens S (2021). Parameters predicting ^18^F-PSMA-1007 scan positivity and type and number of detected lesions in patients with biochemical recurrence of prostate cancer. EJNMMI Res.

[18] Sachpekidis C, Baumer P, Kopka K (2018). ^68^Ga-PSMA PET/CT in the evaluation of bone metastases in prostate cancer. Eur J Nucl Med Mol Imaging.

[19] Afshar-Oromieh A, Vollnberg B, Alberts I (2019). Comparison of PSMA-ligand PET/CT and multiparametric MRI for the detection of recurrent prostate cancer in the pelvis. Eur J Nucl Med Mol Imaging.

[20] Zhou J, Gou Z, Wu R (2019). Comparison of PSMA-PET/CT, choline-PET/CT, NaF-PET/CT, MRI, and bone scintigraphy in the diagnosis of bone metastases in patients with prostate cancer: a systematic review and meta-analysis. Skeletal Radiol.

[21] Hernandez D, Salas D, Gimenez D (2015). Pelvic MRI findings in relapsed prostate cancer after radical prostatectomy. Radiat Oncol.

[22] Liauw SL, Pitroda SP, Eggener SE (2013). Evaluation of the prostate bed for local recurrence after radical prostatectomy using endorectal magnetic resonance imaging. Int J Radiat Oncol Biol Phys.

[23] Stoll M, Stoiber EM, Grimm S (2016). Comparison of safety margin generation concepts in image guided radiotherapy to account for daily head and neck pose variations. PLoS ONE.

[24] Peters N, Wohlfahrt P, Hofmann C (2022). Reduction of clinical safety margins in proton therapy enabled by the clinical implementation of dual-energy CT for direct stopping-power prediction. Radiother Oncol.

[25] Trofimov A, Nguyen PL, Coen JJ (2007). Radiotherapy treatment of early-stage prostate cancer with IMRT and protons: a treatment planning comparison. Int J Radiat Oncol Biol Phys.

[26] Kerkmeijer LGW, Groen VH, Pos FJ (2021). Focal boost to the intraprostatic tumor in external beam radiotherapy for patients with localized prostate cancer: results from the flame randomized phase iii trial. J Clin Oncol.

[27] Salembier C, Villeirs G, De Bari B (2018). ESTRO ACROP consensus guideline on CT- and MRI-based target volume delineation for primary radiation therapy of localized prostate cancer. Radiother Oncol.

