# Supplementary material for: Detecting and Locating the Site of Local Relapse Using 18F-PSMA-1007 Imaging After Primary Treatment of 135 Prostate Cancer Patients—Potential Impact on PSMA-Guided Radiation Therapy
Source: Mol Imaging Biol. 2022 Aug 23;25(2):375–83. doi: 10.1007/s11307-022-01766-6 (PMC10006015; doi:10.1007/s11307-022-01766-6)

**Supplement 1:** template for prostatic fossa in the sagittal plane (lateral left/right, median)

Aal: anastomotic region anterior lateral; Al: anastomotic region lateral; Apl: anastomotic region posterior lateral; Bal: bladder neck anterior lateral; Bl: bladder neck lateral; Bpl: bladder neck posterior lateral; Cal: perivesical anterior lateral; Cl: lateral; SV: seminal vesicle; Aa: anastomotic region anterior; Ac: anastomotic region central; Ap: anastomotic region posterior; Ba: bladder neck anterior; Bc: bladder neck central; Bp: bladder neck posterior; Ca: perivesical anterior; Cc: central; Cp: perivesical posterior


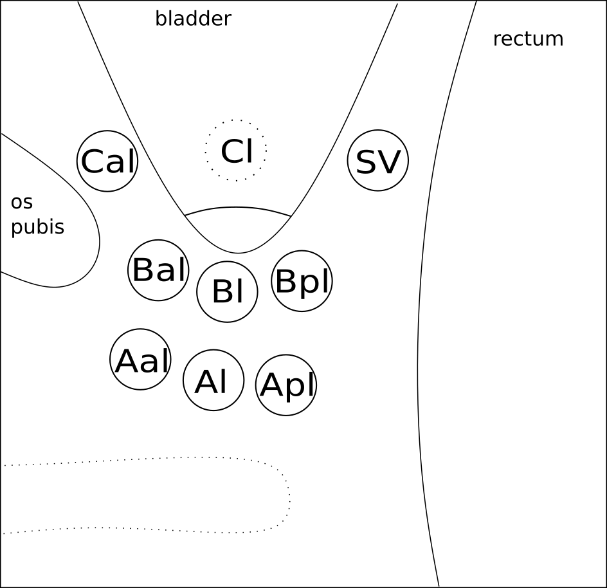

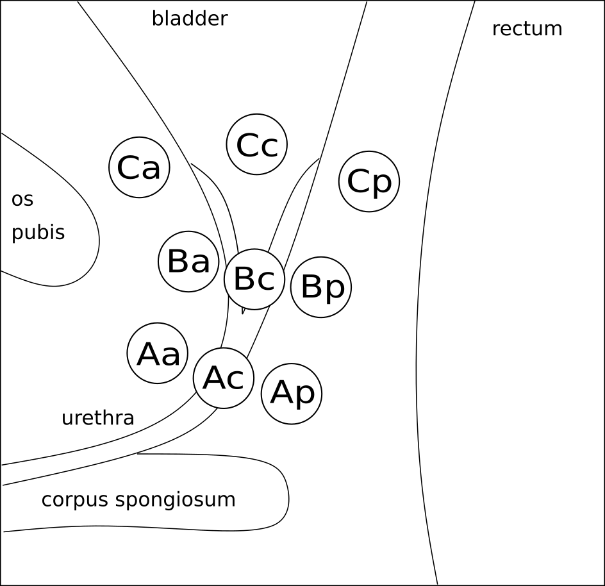

Supplement: Supplementary file 1 — Supplementary file1 (DOCX 99 KB) [file 11307_2022_1766_MOESM1_ESM.docx]
